# A Nation Veering off Course: Implications for Efficacy and Well-Being

**DOI:** 10.3390/bs16030405

**Published:** 2026-03-10

**Authors:** Kristina G. Chamberlin, J. Doris Dai, Hannah F. Ramil, Laura M. Brady, Stephanie A. Fryberg

**Affiliations:** 1 Department of Psychology, Northwestern University, Evanston, IL 60208, USA; fryberg@northwestern.edu; 2 Department of Psychology, University of California, Berkeley, CA 94704, USA; daijdoris@gmail.com; 3 Department of Psychology, University of Michigan, Ann Arbor, MI 48109, USA; hframil@umich.edu; 4 Independent Researcher, Chicago, IL 60606, USA

**Keywords:** efficacy, well-being, coping, COPE, PERMA, national values, political climate

## Abstract

The United States has undergone rapid and, at times, unprecedented political changes in 2025. Recent national polling indicates that many Americans—across political parties—believe that the country is heading in the wrong direction. In a preregistered study with more than 7000 adults residing in the United States, we explored the implications of these widespread concerns for individuals’ psychological functioning. As theorized, individuals who believed that the political climate was worsening and viewed the United States as failing to live up to its core national values experienced lower efficacy, both in terms of their personal ability to influence politics (i.e., individual efficacy) and their confidence in the government to uphold its obligations to the nation and its residents (i.e., government efficacy). In turn, these individuals reported worse overall well-being and less effective coping in response to stressors related to the political climate. These relationships persisted after accounting for the participants’ 2024 presidential vote choice and political party affiliation. Together, these findings suggest that the political turbulence Americans are experiencing exerts a measurable, bipartisan toll on Americans’ psychological and social health.

## 1. Introduction

In recent years, the United States has faced historically high levels of political polarization and instability (Abramowitz & Webster, 2016; Heltzel & Laurin, 2020). By 2025, Americans found themselves in a political climate characterized by an abundance of provocative, divisive, and emotionally charged information. With the rise of 24-hour news cycles and algorithmically driven social media feeds, people in the U.S. have been consistently exposed to content emphasizing political conflict, violence, economic turbulence, and widespread social concerns, such as rising prices, sudden policy reversals, aggressive immigration enforcement, international instability, and cuts to public institutions. Daily, and with little relief, Americans have been forced to make sense of a rapidly changing and unpredictable social and political landscape.

Amid this turmoil, one thing Americans appear to agree upon is how unstable life in the U.S. has become. National polls reveal a rare point of convergence in public opinion: a majority of Americans—including members of both major political parties—believe that the United States is headed in the wrong direction (AP-NORC Center for Public Affairs Research, 2025; Guskin, 2025; Harvard CAPS/Harris Poll, 2025; PRRI, 2025; YouGov, 2025). The percentage of Republicans holding these beliefs rose from 26% in March 2025 to 51% in September 2025, and Democrats reached near consensus, with 91–92% indicating that the nation is headed in the wrong direction (AP-NORC Center for Public Affairs Research, 2025). Moreover, about two-thirds of Americans indicated that both the Republican and Democratic parties are out of touch with the concerns of the average American, and they believe that the current presidential administration has gone too far with policy changes, including levying tariffs, expanding the use of presidential power, and sending federal law enforcement into American cities (AP-NORC Center for Public Affairs Research, 2025; Guskin, 2025).

These perceptions reflect both the long-term erosion of political satisfaction and a uniquely destabilizing political moment. Americans’ trust in government and belief that it will respond to public demands (i.e., external political efficacy) have been declining for decades, reaching historic lows in 2020 and 2024 (American National Election Studies, 2026; Pew Research Center, 2025). At the same time, satisfaction with democracy has fallen globally in recent years, and perceived control—a psychological resource closely linked to efficacy and well-being—was globally suppressed during the early 2020s amid the COVID-19 pandemic (Msetfi et al., 2024; Wike & Fetterolf, 2024). The political climate in 2025 has been further marked by dramatic democratic shifts. Researchers have argued that democratic decline in the U.S. has accelerated since the 2024 presidential election, with autocratic actions and efforts to consolidate executive power creating an unfamiliar, destabilizing, and threatening political environment for everyday Americans (Carrier & Carothers, 2025; Yeung, 2025). This convergence of long-increasing institutional distrust and acute political instability may be further undermining Americans’ confidence in governmental responsiveness and beliefs about one’s own ability to influence politics. Against this backdrop, the present research examines how beliefs about the political climate—such as concerns about the erosion of the nation’s social and political order—may shape Americans’ sense of efficacy and, ultimately, their well-being.

### 1.1. Perceptions of the Current Political Climate

The current political realities have likely implications for Americans’ psychological functioning, as individuals participate in and are fundamentally shaped by the social, political, and cultural contexts in which they live (Markus & Kitayama, 2010). People make sense of the world through the prevailing narratives they encounter in their social contexts, which guide how they interpret the past and present, and how they envision the future (Kelly, 1955). Individuals can glean information about areas of progress or decline from current events, concurrently drawing conclusions about the state of the nation, including whether it is succeeding in—or failing to live up to—its goals and values.

Faced with a constant flow of information about rapid and widespread political change, Americans today may have come to believe that societal functioning—and perhaps the very nation itself—is in decline. Indeed, national polling indicates that Americans are sensing decays in societal conditions across several political domains, including the economy, government performance, immigration, and international relations (PRRI, 2025). Moreover, people perceive that the nation’s core moral and ideological values are deteriorating. These core American values, such as fairness, equality, the rule of law, democratic norms, and social mobility, have long served as benchmarks for what the nation is and what it aspires to be (Beasley, 2001; Lipset, 1996). When people witness repeated violations of these core values—such as the uneven application of laws, leaders abusing executive power, and economic systems failing to provide mobility—they may start to believe that the system no longer operates as intended (Ariely & Uslaner, 2017; Schnaudt et al., 2021; van der Meer & Hakhverdian, 2016). Indeed, the *Democracy for All Project* (Gallup & Charles F. Kettering Foundation, 2025) found that while approximately two-thirds of Americans believe that democracy is the best form of government, 51% reported that democracy in the U.S. is doing ‘poorly’ to ‘very poorly’ right now, suggesting that most Americans feel that the nation is failing to live up to its ideal system of governance.

### 1.2. Implications of the Political Climate for Efficacy

Living in a social and political environment that feels unstable or deteriorative can undermine individuals’ beliefs in their ability to influence the world around them and diminish their confidence that existing systems are working effectively toward a positive future (Alessandro et al., 2021; Hetherington, 1998; Hetherington & Rudolph, 2015; Magni, 2017; Miller & Listhaug, 1999). Accordingly, we theorize that the current political climate may diminish Americans’ sense of efficacy in two important ways.

First, immersed in narratives of decline paired with rapid, widespread political changes, people may come to feel that they have little personal control over the social and political upheavals that are unfolding in the U.S. (Campbell et al., 1954; Craig et al., 1990; Olsen, 1969; Seeman, 1959). We refer to this phenomenon as a reduction in *individual efficacy.* Rapid political shifts limit individuals’ opportunities to voice opinions and shape political change in meaningful ways. Under these conditions, individuals might deduce that the political system is too dysfunctional for individual participation to make a difference (Ariely & Uslaner, 2017; Balch, 1974; Campbell et al., 1954; Niemi et al., 1991). Indeed, research indicates that individual-level efficacy diminishes in the face of institutional drift or disorder—particularly during national crises—leading to withdrawal from civic engagement (Hetherington & Rudolph, 2015; Magni, 2017).

Second, given the prevailing perceptions of the current political climate revealed through polling, individuals may feel that their government leaders have disregarded their responsibilities to protect and defend the interests and values of the nation and its people (Citrin & Stoker, 2018; Friedrichs, 1980; Gil de Zúñiga et al., 2017; Niemi et al., 1991). We refer to this belief as a reduction in *government efficacy.* When individuals witness issues like inflation, government budget deficits, and deteriorating public safety, they may conclude that their government lacks either the capacity or the willingness to meet the public interest. Additionally, if individuals believe that core values such as fairness and accountability are being compromised, they are likely to view government officials as untrustworthy stewards of the political system. Previous research has shown that confidence in the government declines sharply when people begin to question its effectiveness and the extent to which it values integrity, openness, and fairness (Alessandro et al., 2021; Hetherington, 1998; Miller & Listhaug, 1999).

### 1.3. Reduced Efficacy Affords Poorer Well-Being

Declines in efficacy are linked to adverse psychological well-being. During times of instability or uncertainty, it is essential for individuals to believe that they and their governing systems can improve outcomes, as these beliefs help them cope with stress, make sense of events, and maintain psychological resilience (Bandura, 1997; Collins, 2025; Lachman & Weaver, 1998; Skaff, 2007). When people feel a diminished sense of personal control, they experience greater feelings of helplessness and disengagement (Magaletta & Oliver, 1999). Similarly, when individuals perceive their government as unresponsive, incompetent, or untrustworthy in improving outcomes, they report higher levels of cynicism, frustration, and emotional exhaustion (Hetherington & Rudolph, 2015; Miller & Listhaug, 1999). Recent work has demonstrated that institutional distrust and perceptions of political dysfunction amid instability are linked to lower life satisfaction and increased collective anxiety across the globe (Aassve et al., 2024; El Khoury-Malhame et al., 2024; Oishi et al., 2018). Together, these findings suggest that diminished efficacy leaves individuals more vulnerable to stress, hopelessness, and declines in overall well-being.

### 1.4. The Current Work

Prior evidence suggests that, to the extent that individuals perceive declines in societal conditions and in the core values of the nation, they may experience reduced individual and government efficacy and, in turn, report depressed well-being. The current preregistered study uses this framework to assess how Americans across the political spectrum are navigating the U.S. political climate in 2025 (see Supplementary Materials for the pre-registration). Using data from a national sample with more than 7000 participants, we examined the relationships between the individuals’ perceptions of the political climate, beliefs about whether the U.S. is living up to its core values, individual and government efficacy, and well-being.

**Figure 1:**
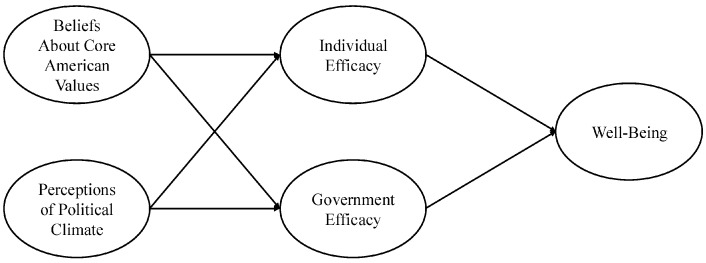
Proposed conceptual model.

We hypothesized that, to the extent that Americans feel that societal conditions—such as the economy, immigration policy, international relations, and the like—are declining and believe the U.S. is failing to live up to its core national values (e.g., democracy, equality, and rule of law), they would report feeling less able to influence the political process and have less confidence in the government. In turn, these individuals would report worse well-being (see Figure 1 for conceptual model). We also tested whether these hypothesized relationships remained after controlling for the participants’ political inclinations, including their voting patterns and political party affiliations.

## 2. Materials and Methods

### 2.1. Participants

This research was approved by the Institutional Review Board at Northwestern University (STU00223835, 9 April 2025). We recruited a sample of adults residing in the U.S. (*N* = 8031) via Qualtrics Research Panels. We utilized custom sampling quotas to collect roughly equal proportions of Trump voters and Harris voters and to ensure that we sampled participants from each U.S. state. Before participating in the survey, participants were provided with a description of the study and were asked to indicate electronically whether they consented to participate. Participants who consented to participate completed an online survey and received compensation commensurate with the payment methods and standards of their panel membership. Prior to analysis, participants were excluded sequentially if they met the following criteria: (1) their data indicated long string responding (*n* = 49); (2) their total duration was beyond the upper or lower 2.5% of the sample’s duration distribution (*n* = 394); (3) their intra-individual response variability (IRV) was exceptionally high or low, indicating highly random or straight-line responding, respectively (*n* = 372); and (4) their open-ended responses indicated obvious use of AI or chatbots based on a hidden prompt (*n* = 1). Our final sample included 7215 participants (90% of the original sample) between the ages of 18 and 96 (*M* = 53.01, *SD* = 17.23). Participants from all fifty states and the District of Columbia were represented. Additional demographics for the final sample are summarized in Table 1.

### 2.2. Measures and Procedures

The measures utilized in this study comprised a subsample of the measures included in a survey conducted between May and October 2025 (see Supplementary Materials for the full measures, including instructions, items, and rating scales). Measures were organized into thematic blocks. The order of blocks and measures within blocks was randomized.

The factor structure of each measure was evaluated using exploratory factor analysis with oblimin rotation (EFA; for new measures developed by the research team) or confirmatory factor analysis (CFA; for established measures). For the EFAs, suitability for EFA was assessed using the Kaiser–Meyer–Olkin (KMO) measure and Bartlett’s test of sphericity, and the number of factors to test was determined by the number of eigenvalues above 1.00 and parallel analysis (see Supplementary Materials). For the CFAs and EFAs, model fit was assessed using CFI (excellent ≥ 0.95, acceptable = 0.90–0.94) and RMSEA (excellent ≤ 0.05, acceptable = 0.06–0.08). Convention aims for primary loadings greater than or equal to 0.40, cross-loadings below 0.30, communalities at or above 0.40, and factor correlations below 0.70 (Brown, 2015; Kline, 2016; Hu & Bentler, 1999; Fabrigar & Wegener, 2012).

**Perceptions of the current political climate.** To assess perceptions of the current political climate, we developed 19 items reflecting prominent topics dominating news headlines and U.S. political discourse at the time of survey development, such as the strength of the economy, foreign relations, immigration policy, civil rights, public health, and other topics. Participants were asked to rate whether they thought things were getting better or worse in each area on a seven-point Likert scale from 1 = *getting dramatically worse* to 7 = *getting dramatically better*. An EFA found that a one-factor structure produced an adequate fit to the data (CFI = 0.95, RMSEA = 0.08), accounting for 72.9% of the variance. Each item loaded well (0.76 to 0.91), and communalities were good (0.58 or greater; *α* = 0.98, *M* = 3.27, *SD* = 1.63).

**Beliefs about core American values.** We developed a 19-item measure to assess the extent to which participants believed that the U.S. was living up to its core national values. Items reflected common values people associate with the United States, such as equality, democracy, individual freedom, and self-reliance (e.g., Lipset, 1996). Participants used a 1 = *strongly disagree* to 6 = *strongly agree* scale to rate (1) how much they believed that the U.S. is living up to each value and (2) whether each concept should be a core American value. To account for individual differences in the level of importance assigned to each concept, we weighted each “living up to” rating by its corresponding “should be” rating and treated each weighted score as an indicator of a latent construct representing perceived performance on core national values. An EFA indicated that a one-factor model demonstrated a good fit to the data (CFI = 0.97, RMSEA = 0.05), accounting for 61.5% of the variance. All items loaded well (0.66 to 0.84), and communalities were acceptable (0.43 or greater; *α* = 0.97, *M* = 3.35, *SD* = 1.08).

**Efficacy**. We used two measures to assess changes in perceived efficacy related to the political climate (adapted; American National Election Studies, 2025; Niemi et al., 1991; Gil de Zúñiga et al., 2017). The first measure reflected individual efficacy (3 items; e.g., “Since Donald Trump’s re-election, my say/voice in the government has…” rated from 1 = *significantly decreased* to 7 = *significantly increased*) and the second measure reflected government efficacy (3 items, e.g., “Since Donald Trump’s re-election, the government is _____ to making decisions based on what the citizens want” rated from 1 = *significantly less committed* to 7 = *significantly more committed*). Items were rated on 7-point scales (additional scale point labels are available in the Supplementary Materials) and reverse-coded as needed. We utilized CFA to assess the structure of each efficacy measure. Because these models were fully saturated, overall fit could not be assessed, but we did attend to factor loadings. Loadings were generally acceptable for individual efficacy (0.53 to 0.88) and government efficacy (0.33 to 0.95). One government efficacy item fell below the conventional threshold of 0.40, but in order to maintain a minimum of three items per measure for latent variable modeling, we chose to keep the item. The internal reliabilities for individual efficacy (*α* = 0.78, *M* = 3.84, *SD* = 1.61) and government efficacy (*α* = 0.76, *M* = 3.25, *SD* = 1.77) were acceptable.

**Well-being.** We measured well-being in two ways. First, we considered how individuals were coping with current political stressors (Brief COPE Scale; Carver, 1997). Second, we assessed their overall sense of well-being across several domains (e.g., emotions, relationships, health, etc.; PERMA-Profiler, Butler & Kern, 2016).

***Brief COPE Scale.*** To assess coping, participants completed the Brief COPE Scale (“COPE”), which included 28 items (reflecting 14 two-item subscales) assessing various ways of coping with stress. We modified the COPE instructions to ask participants to reflect on how they responded to stress related to the current political climate. Participants reviewed a list of coping behaviors and rated how much they engaged in each behavior on the following scale: 1 = *I haven’t been doing this at all*, 2 = *I’ve been doing this a little bit*, 3 = *I’ve been doing this a medium amount*, 4 = *I’ve been doing this a lot.* We performed a confirmatory factor analysis (CFA) where each coping strategy was loaded onto its own latent variable (i.e., a 14-factor model with two indicators apiece). The model demonstrated a good fit to the data, CFI = 0.97, TLI = 0.96, RMSEA = 0.04, SRMR = 0.03, and all factor loadings exceeded the conventional threshold of 0.40 (0.64 to 0.95).

The 14 COPE subscales include self-distraction (*α* = 0.62, *M* = 2.41, *SD* = 0.93), denial (*α* = 0.73, *M* = 1.79, *SD* = 0.92), substance use (*α* = 0.92, *M* = 1.51, *SD* = 0.88), behavioral disengagement (*α* = 0.75, *M* = 1.67, *SD* = 0.85), venting (*α* = 0.68, *M* = 2.11, *SD* = 0.89), self-blame (*α* = 0.80, *M* = 1.87, *SD* = 0.98), active coping (*α* = 0.71, *M* = 2.59, *SD* = 0.90), use of emotional support (*α* = 0.79, *M* = 2.12, *SD* = 0.94), use of instrumental support (*α* = 0.83, *M* = 2.02, *SD* = 0.94), positive reframing (*α* = 0.74, *M* = 2.39, *SD* = 0.91), planning (*α* = 0.80, *M* = 2.52, *SD* = 0.97), humor (*α* = 0.82, *M* = 2.06, *SD* = 0.97), acceptance (*α* = 0.67, *M* = 2.70, *SD* = 0.88), and religion (*α* = 0.85, *M* = 2.40, *SD* = 1.12).

For ease of interpretation, self-distraction, denial, substance use, behavioral disengagement, venting, and self-blame are often grouped together as “maladaptive” coping strategies, while active coping, use of emotional or instrumental support, positive reframing, planning, humor, acceptance, and religion are grouped as “adaptive” coping strategies (Meyer, 2001). However, psychometrically, it is most valid to evaluate each construct individually (Rodrigues et al., 2022). Therefore, we assessed each COPE construct individually and considered overall patterns of maladaptive and adaptive coping strategies when drawing conclusions. Higher engagement in maladaptive coping strategies typically indicates greater distress and lower well-being, whereas higher engagement in adaptive coping strategies indicates less distress and greater well-being (Kato, 2013).

***PERMA-Profiler.*** To assess overall well-being, participants completed the PERMA-Profiler (“PERMA”), which includes 23 items measuring nine aspects of well-being. Participants rated each item on a scale from 0 to 10 (see specific scale points in Supplementary Materials). Positively-valenced subscales included Positive Emotion (three items; *α* = 0.89, *M* = 6.46, *SD* = 2.37); Engagement (three items; *α* = 0.60, *M* = 6.73, *SD* = 1.86); Relationships (three items; *α* = 0.84, *M* = 6.69, *SD* = 2.46); Meaning (three items; *α* = 0.91, *M* = 6.71, *SD* = 2.46); Accomplishment (two items;^1^ *α* = 0.63, *M* = 6.93, *SD* = 2.03); Health (three items; *α* = 0.92, *M* = 6.33, *SD* = 2.35); and Happiness (one item; *M* = 6.75, *SD* = 2.59), with higher scores indicating greater well-being. Negatively-valenced subscales included Negative Emotion (three items; *α* = 0.82, *M* = 4.31, *SD* = 2.50) and Loneliness (one item; *M* = 4.36, *SD* = 3.31), with higher scores indicating poorer well-being. A CFA reflecting this 9-factor structure indicated that the model provided a good fit to the data, CFI = 0.96, TLI = 0.95, RMSEA = 0.06, SRMR = 0.04. Although one indicator of the Engagement construct fell below the conventional loading threshold of 0.40 (0.34), we retained the item, as it was part of a validated measure.

**Covariates.** Individuals from different sides of the political spectrum might differ in their satisfaction with the state of the union, which could have implications for their feelings of efficacy and well-being. To account for these variations, we assessed the participants’ voting patterns and political party affiliations and included these measures as covariates in our analyses. This procedure allowed us to examine whether the hypothesized effects remained after accounting for the influence of the participants’ political inclinations. Descriptive statistics of our key model variables by voter type and political party, as well as additional analyses including political ideology, identity, and identity centrality covariates, are included in the Supplementary Materials.

***Voter type.*** Participants were asked to select who they voted for in the 2024 presidential election. Options included *Donald Trump (Republican Party); Kamala Harris (Democratic Party); Jill Stein (Green Party); Chase Oliver (Libertarian Party); Robert F. Kennedy, Jr. (Independent); another candidate* (write-in); *ineligible to vote;* or *eligible to vote, but did not vote*. In our analyses, we created dummy codes for Harris voters; third-party voters (combining Stein, Oliver, Kennedy, and another candidate); ineligible voters; and eligible non-voters, leaving Trump voters as the comparison group.

***Political party affiliation.*** Participants indicated their party affiliation by selecting *Republican, Democrat, independent/unaffiliated, Libertarian, Green Party, Tea Party, Socialist, Democratic Socialist,* or *another political party* (write-in). For analyses, we created dummy codes for Democrats, independents, and third-party affiliates (combining Libertarian, Green Party, Tea Party, Socialist, Democratic Socialist, and another political party), with Republicans as the comparison group.

## 3. Results

### 3.1. Analytic Plan

To test our hypothesis that negative perceptions of the current political climate and beliefs that the U.S. is failing to live up to its core national values would be associated with reduced efficacy and, in turn, diminished well-being, we implemented a two-step procedure commonly used in structural equation modeling (Anderson & Gerbing, 1988; Kline, 2016). First, we utilized CFA to evaluate the measurement model for our latent variables. The purpose of the measurement model is to verify the latent structure of the measures and to assess convergent and discriminant validity by attending to the correlations between latent variables. Second, we constructed a structural equation model (SEM) to test the hypothesized relationships between the latent variables and assess whether our proposed model was supported (i.e., the “base” model). After completing the two-step procedure, we added covariates to the SEM to examine the stability of the relationships between the latent variables after accounting for the participants’ political inclinations (i.e., the “covariate” model). We conducted parallel sets of CFA and SEM analyses to test our hypothesized model in relation to overall well-being (i.e., PERMA) and coping behaviors (i.e., COPE). All analyses were performed using the *lavaan* package in R (Rosseel, 2012). All models utilized maximum likelihood (ML) estimation and ran 5000 bootstrap resamples to generate standard errors and 95% confidence intervals. All models were evaluated in accordance with Hu and Bentler’s (1999) model fit guidelines, which suggest that a model fits the data well when it produces a nonsignificant Chi-squared test of model fit; CFI and TLI of 0.95 and above; RMSEA of 0.06 or below; and SRMR of 0.08 or below. However, given that these are heuristics rather than firm thresholds, we holistically evaluated the model fit indices and their closeness to these values when judging model fit (van Laar & Braeken, 2022).

### 3.2. Well-Being: PERMA

**Measurement model.** The PERMA model included latent variables for perceptions of the political climate; beliefs about core American values; individual efficacy; government efficacy; and each PERMA construct (Positive Emotion; Engagement; Relationships; Meaning; Accomplishment; Health; Negative Emotion). Because the Happiness and Loneliness PERMA constructs were single item measures, they were included as observed variables rather than latent variables. All latent constructs, as well as Happiness and Loneliness, were allowed to correlate. The measurement model demonstrated acceptable fit to the data, *χ*^2^ (2003) = 25,592.49, *p* < 0.001; CFI = 0.95; TLI = 0.94; RMSEA = 0.04; SRMR = 0.04, and variables were generally correlated in expected ways (see Supplementary Materials, Figure S1 for details). Therefore, the measurement model provided good support to proceed with SEM analysis. However, of note, individual and government efficacy were highly correlated (*r* = 0.85), introducing potential issues with multicollinearity (Brown, 2015; Kline, 2016), so we ran separate SEMs for individual and government efficacy.

**SEM: base models.** In the first model, we regressed each dimension of PERMA onto individual efficacy and regressed individual efficacy onto perceptions of the political climate and beliefs about core American values. In the second model, we ran the same model but replaced individual efficacy with government efficacy. In both models, perceptions of the political climate and beliefs about core American values were allowed to correlate, as were the PERMA variables^2^. For each model, we assessed whether the theorized pathways were supported by multiplying (A) each association between perceptions of the political climate and beliefs about core American values with efficacy by (B) each association between efficacy and well-being (i.e., the indirect effects).

**Figure 2:**
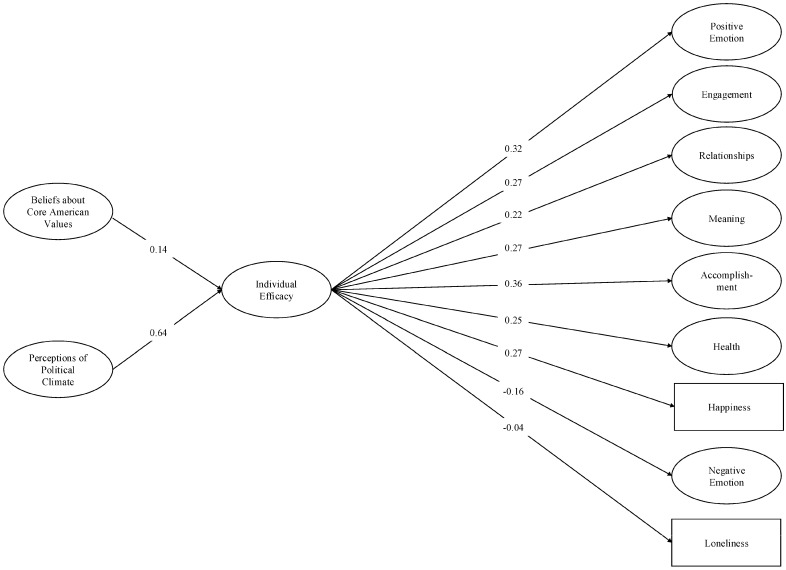
Structural equation model: Individual efficacy and PERMA (base model). *Note.* Single-headed arrows represent standardized path coefficients. Statistically significant relationships (*p* < 0.05) are represented by solid lines. For ease of interpretation, we excluded any covariances between the latent constructs from the figure. These covariances as well as additional statistics (e.g., unstandardized estimates, factor loadings, confidence intervals) are in the Supplementary Materials.

**Figure 3:**
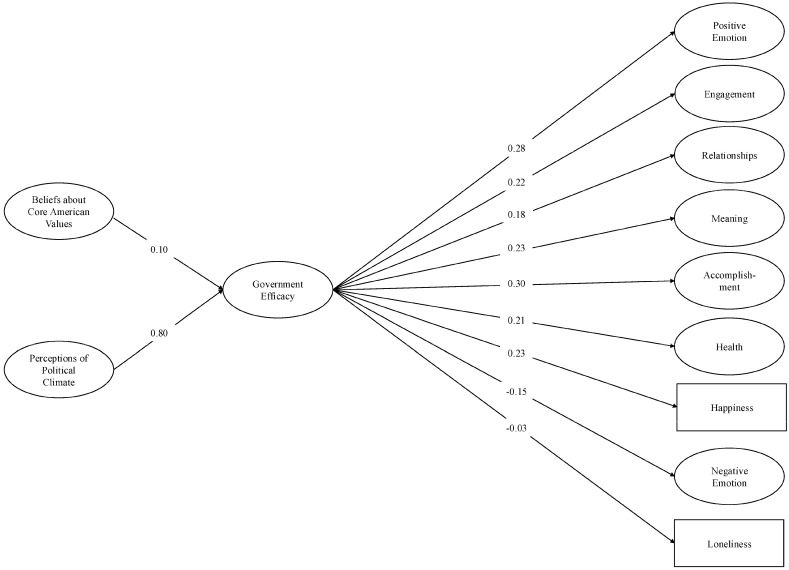
Structural equation model: Government efficacy and PERMA (base model). *Note.* Single-headed arrows represent standardized path coefficients. Statistically significant relationships (*p* < 0.05) are represented by solid lines. For ease of interpretation, we excluded any covariances between the latent constructs from the figure. These covariances as well as additional statistics (e.g., unstandardized estimates, factor loadings, confidence intervals) are in the Supplementary Materials.

The models demonstrated acceptable fit to the data: for individual efficacy, *χ*^2^ (1844) = 23,605.98, *p* < 0.001; CFI = 0.95; TLI = 0.94; RMSEA = 0.04; SRMR = 0.06 (see Figure 2), and for government efficacy, *χ*^2^ (1844) = 24,651.19, *p* < 0.001; CFI = 0.95; TLI = 0.94; RMSEA = 0.04; SRMR = 0.06 (see Figure 3). As predicted, individuals with more negative perceptions of the political climate and who believed that the United States was failing to live up to its core values reported lower levels of individual and government efficacy. Each type of efficacy was associated with diminished well-being in each of the nine PERMA domains (i.e., lower scores on the positively-valenced measures; higher scores on the negatively-valenced measures). Moreover, each of the indirect effects was significant (see Table 2 and Table 3). These results support our theorizing that people who perceived the political climate to be worsening and believed that the U.S. was not living up to its core national values also felt that their own political voice and action had become less important and felt less confident that the government had the people’s interests in mind. In turn, they reported worse overall well-being, such as worse relationships, a diminished sense of meaning and accomplishment, and worse health.

**Figure 4:**
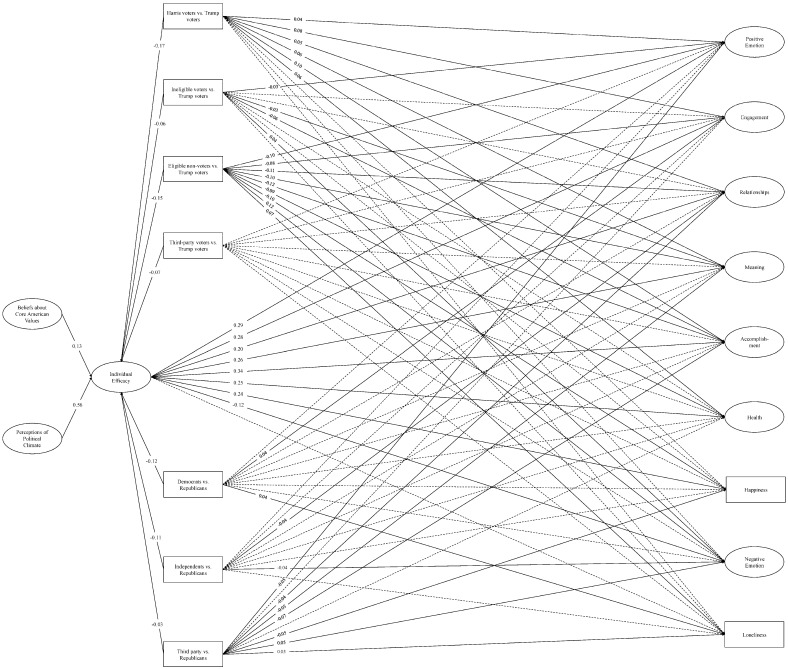
Structural equation model: Individual efficacy and PERMA (covariate model). *Note.* Single-headed arrows represent standardized path coefficients. Statistically significant relationships (*p* < 0.05) are represented by solid lines and nonsignificant relationships (*p* ≥ 0.05) are represented by dotted lines. For ease of interpretation, we excluded any covariances between the latent constructs from the figure as well as nonsignificant path coefficients. Additional statistics (e.g., covariances, unstandardized estimates, factor loadings, confidence intervals) are in the Supplementary Materials.

**Figure 5:**
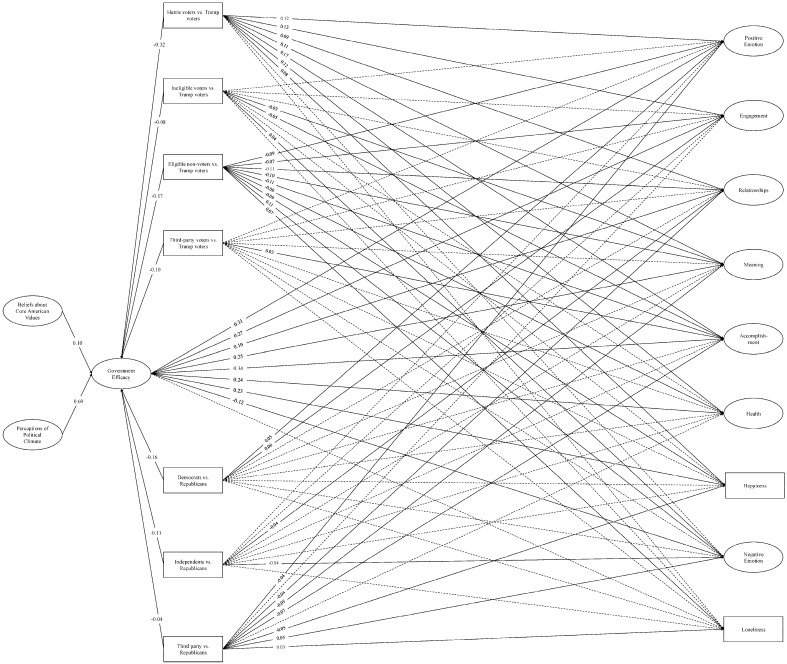
Structural equation model: Government efficacy and PERMA (covariate model). *Note.* Single-headed arrows represent standardized path coefficients. Statistically significant relationships (*p* < 0.05) are represented by solid lines and nonsignificant relationships (*p* ≥ 0.05) are represented by dotted lines. For ease of interpretation, we excluded any covariances between the latent constructs from the figure as well as nonsignificant path coefficients. Additional statistics (e.g., covariances, unstandardized estimates, factor loadings, confidence intervals) are in the Supplementary Materials.

**SEM: covariate models.** We then re-ran the SEMs, covarying voter type and political party affiliation with each of the efficacy and PERMA variables. The individual efficacy model yielded an adequate fit to the data, *χ*^2^ (2215) = 29,897.70, *p* < 0.001; CFI = 0.93; TLI = 0.93; RMSEA = 0.04; SRMR = 0.09 (see Figure 4), as did the government efficacy model, *χ*^2^ (2215) = 30,167.49, *p* < 0.001; CFI = 0.94; TLI = 0.93; RMSEA = 0.04; SRMR = 0.09 (see Figure 5).

Generally, Harris voters reported better well-being than Trump voters in the positively-valenced domains, whereas eligible non-voters reported worse well-being than Trump voters across all nine outcomes. Ineligible voters also reported worse well-being than Trump voters on some outcomes (e.g., Meaning, Accomplishment, Negative Emotion) but otherwise did not show consistent differences. Across models, third-party and Trump voters did not demonstrate any consistent differences in well-being. For political party affiliation, Democrats’ and independents’ PERMA scores differed from Republicans in limited and directionally inconsistent ways, but third-party affiliates generally reported worse well-being than Republicans. In terms of individual and government efficacy, Harris voters, ineligible voters, eligible non-voters, and third-party voters alike felt less efficacious than Trump voters, and Democrats, independents, and third-party affiliates felt less efficacious than Republicans.

We then examined whether the relationships and indirect effects from our base models remained after accounting for political inclinations. As before, perceiving the political climate to be worsening and believing that the U.S. is failing to live up to its core values were associated with less individual and government efficacy. Reduced efficacy of either kind was associated with worse well-being in every domain except Loneliness (nonsignificant). Accordingly, each indirect effect from the base models—barring those involving Loneliness—remained significant in the covariate models (see Table 4 and Table 5).

In summary, people who felt that the political climate was worsening and believed that the U.S. was failing to live up to its core values also showed an inhibited sense that they could personally make a political impact, as well as a decreased sense that the government was able or willing to meet the needs of its constituents. In turn, these individuals reported worse well-being across PERMA measures, including their relationships, sense of accomplishment and meaning, health, and other domains. The indirect effects suggest that reduced efficacy may help explain why negative perceptions of the political climate and of the nation’s functioning predict poorer well-being. However, additional experimental evidence is needed to understand the causal direction of this relationship. With the exception of Loneliness, these patterns remained robust after accounting for the voting patterns and party affiliations of our participants, suggesting that our proposed model was relevant to individual functioning across the political spectrum.

### 3.3. Well-Being: COPE

**Measurement model.** In the COPE model, we included latent variables for perceptions of the political climate, beliefs about core American values, both forms of efficacy, and each COPE construct. All latent variables were allowed to correlate. The measurement model demonstrated an acceptable fit to the data, *χ*^2^ (2331) = 24,792.82, *p* < 0.001; CFI = 0.95; TLI = 0.94; RMSEA = 0.04; SRMR = 0.04, so we proceeded to the SEM analysis (see Figure S2 in Supplementary Materials for more details on latent factor structures and correlations between latent variables). As before, the two forms of efficacy were highly correlated in the measurement model (*r* = 0.85)*,* so we ran separate SEMs involving individual and government efficacy.

**SEM: base models.** In the first SEM, we regressed each COPE construct onto individual efficacy and regressed individual efficacy onto perceptions of the political climate and beliefs about core American values. In the second SEM, we ran the same model, but individual efficacy was replaced with government efficacy. In each model, perceptions of the climate and core American values were correlated, as were each of the COPE variables. We calculated indirect effects by multiplying (A) each of the relationships between perceptions of the political climate and beliefs about core American values and both forms of efficacy by (B) each of the relationships between individual and government efficacy and each COPE outcome.

**Figure 6:**
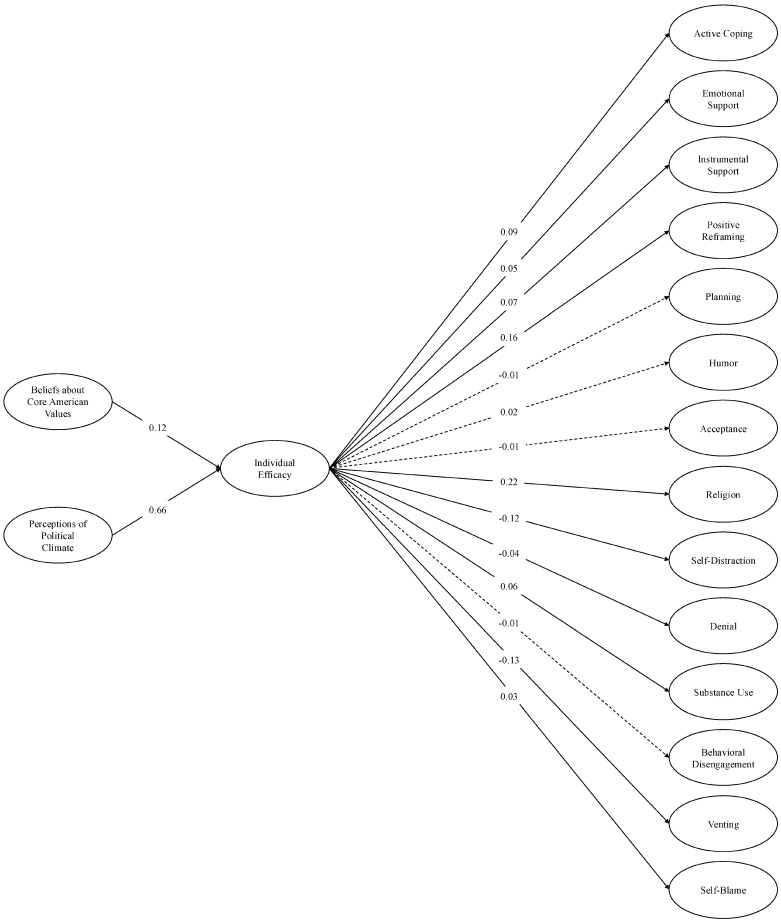
Structural equation model: Individual efficacy and COPE (base model). *Note.* Single-headed arrows represent standardized path coefficients. Statistically significant relationships (*p* < 0.05) are represented by solid lines and nonsignificant relationships (*p* ≥ 0.05) are represented by dotted lines. For ease of interpretation, we excluded any covariances between the latent constructs from the figure. These covariances as well as additional statistics (e.g., unstandardized estimates, factor loadings, confidence intervals) are in the Supplementary Materials.

**Figure 7:**
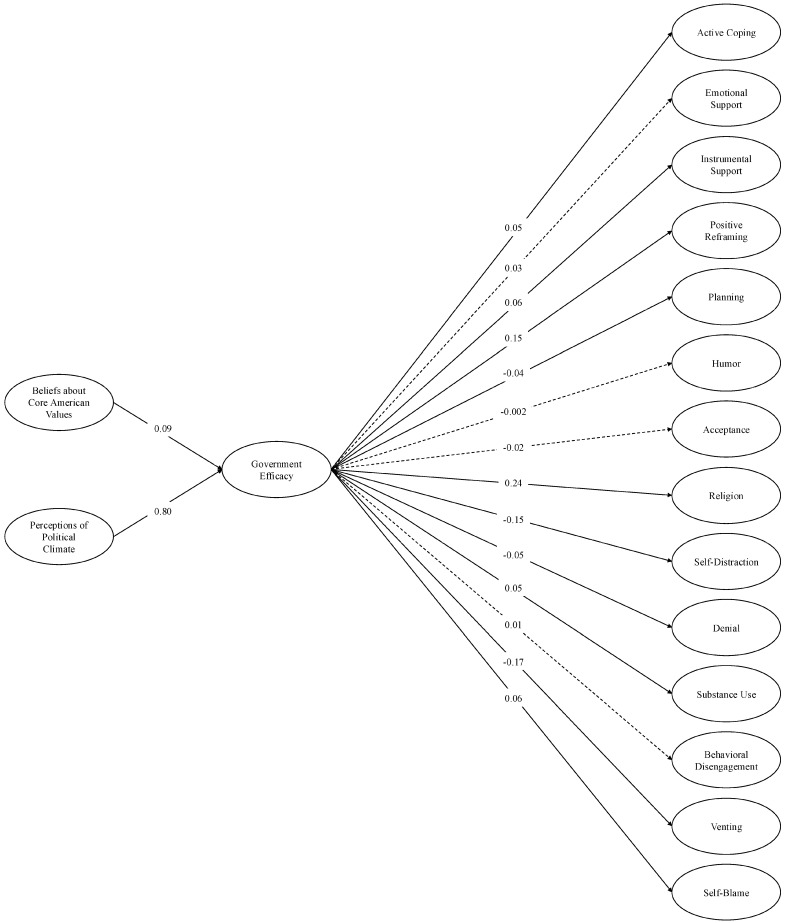
Structural equation model: Government efficacy and COPE (base model). *Note.* Single-headed arrows represent standardized path coefficients. Statistically significant relationships (*p* < 0.05) are represented by solid lines and nonsignificant relationships (*p* ≥ 0.05) are represented by dotted lines. For ease of interpretation, we excluded any covariances between the latent constructs from the figure. These covariances as well as additional statistics (e.g., unstandardized estimates, factor loadings, confidence intervals) are in the Supplementary Materials.

Model fit was acceptable for both the individual efficacy model, *χ*^2^ (2169) = 22,772.58, *p* < 0.001; CFI = 0.95; TLI = 0.94; RMSEA = 0.04; SRMR = 0.04 (see Figure 6), and the government efficacy model, *χ*^2^ (2169) =23,351.61, *p* < 0.001; CFI = 0.95; TLI = 0.94; RMSEA = 0.04; SRMR = 0.04 (see Figure 7). Consistent with the PERMA analyses, more negative perceptions of the political climate and believing the U.S. was failing to live up to its core values were again associated with less individual and government efficacy. Lower individual and government efficacy were each associated with reduced engagement in adaptive coping strategies (most consistently active coping, instrumental support, positive reframing, and religion). They were also associated with more engagement in some maladaptive coping strategies—especially denial, self-distraction, and venting—but less engagement in other maladaptive strategies, such as substance use or self-blame.

Accordingly, in the individual efficacy model, the indirect effects were reliably significant—and in the expected direction—for five of the eight adaptive coping strategies (active coping, emotional and instrumental support, positive reframing, religion), and for three of the six maladaptive strategies (denial, self-distraction, venting; see Table 6). In the government efficacy model, the indirect effects were reliably significant and in the expected direction for four of the eight adaptive strategies (active coping, instrumental support, positive reframing, and religion) and three of the six maladaptive strategies (denial, self-distraction, venting; see Table 7). Together, and consistent with our hypothesized model, the indirect effects suggest that efficacy may help explain the link between perceptions of U.S. societal conditions and the use of various coping strategies. Namely, perceiving poorer social conditions and failure to live up to nation-defining standards were associated with reduced individual and government efficacy, each of which related to decreased engagement in some adaptive coping strategies and increased engagement in some maladaptive strategies.

**Figure 8:**
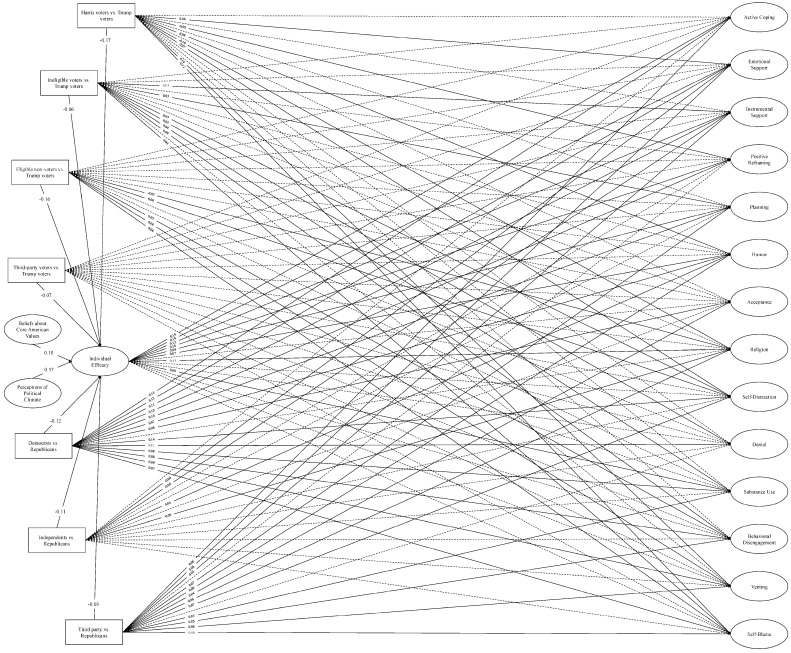
Structural equation model: Individual efficacy and COPE (covariate model). *Note.* Single-headed arrows represent standardized path coefficients. Statistically significant relationships (*p* < 0.05) are represented by solid lines and nonsignificant relationships (*p* ≥ 0.05) are represented by dotted lines. For ease of interpretation, we excluded any covariances between the latent constructs from the figure as well as nonsignificant path coefficients. Additional statistics (e.g., covariances, unstandardized estimates, factor loadings, confidence intervals) are in the Supplementary Materials.

**Figure 9:**
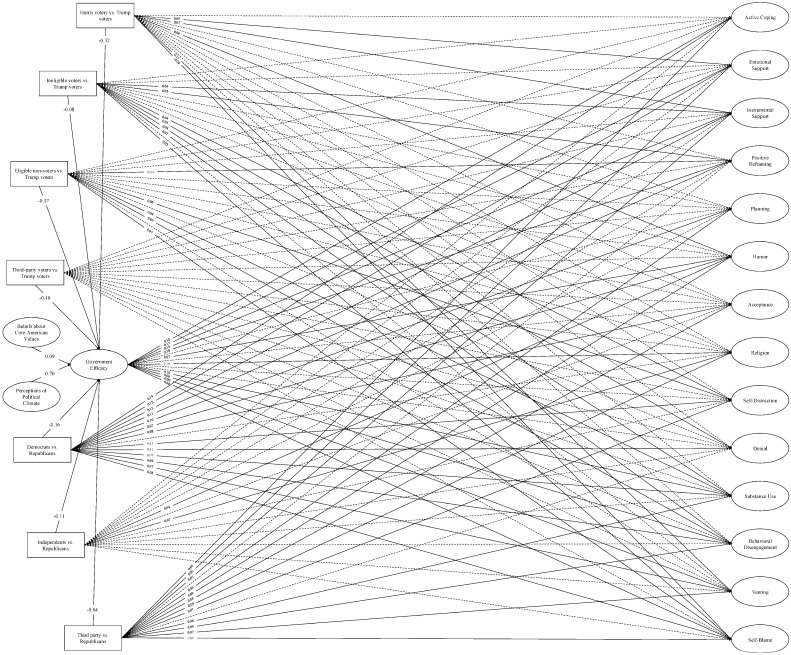
Structural equation model: Government efficacy and COPE (covariate model). *Note.* Single-headed arrows represent standardized path coefficients. Statistically significant relationships (*p* < 0.05) are represented by solid lines and nonsignificant relationships (*p* ≥ 0.05) are represented by dotted lines. For ease of interpretation, we excluded any covariances between the latent constructs from the figure as well as nonsignificant path coefficients. Additional statistics (e.g., covariances, unstandardized estimates, factor loadings, confidence intervals) are in the Supplementary Materials.

**SEM: covariate models.** We then re-ran the SEMs with the voter type and political party affiliation covariates. Model fit was adequate for both the individual efficacy model, *χ*^2^ (2547) = 28,776.74, *p* < 0.001; CFI = 0.93; TLI = 0.93; RMSEA = 0.04; SRMR = 0.08 (see Figure 8), and the government efficacy model, *χ*^2^ (2547) = 28,838.44, *p* < 0.001; CFI = 0.94; TLI = 0.93; RMSEA = 0.04; SRMR = 0.08 (see Figure 9).

Overall, as in the PERMA models, the COPE models revealed that Harris voters, ineligible voters, eligible non-voters, and third-party voters felt less individual and government efficacy than Trump voters. Democrats, independents, and third-party affiliates also felt less individual and government efficacy than Republicans.

Voting patterns were also associated with coping. When significant differences arose, ineligible voters and eligible non-voters engaged in maladaptive strategies more than Trump voters (e.g., self-distraction, behavioral disengagement, self-blame). Trump and Harris voters showed few differences in the use of maladaptive coping strategies, and third-party voters showed no differences compared to Trump voters. Voter type demonstrated few differences in the use of adaptive strategies, but when significant differences emerged, Harris voters and ineligible voters generally used more adaptive strategies than Trump voters.

Finally, for political party affiliation, overall patterns suggested that with limited exceptions, Democrats and third-party affiliates tended to engage in more adaptive and maladaptive coping strategies than Republicans, while independents differed from Republicans in inconsistent and limited ways.

We then examined whether the indirect effects from our base COPE models remained after accounting for voter type and political party (see Table 8 and Table 9). In the covariate model involving individual efficacy, the significant indirect effects for the adaptive strategies (active coping, emotional and instrumental support, positive reframing, and religion) remained significant. In contrast to the base model, the indirect effects for planning and humor also became significant in the covariate model. These results suggest that the relationships between perceptions of the political climate, beliefs about core American values, individual efficacy, and adaptive coping were largely independent of the effects of political inclinations. Conversely, for the maladaptive strategies, most of the indirect effects from the base model were nonsignificant in the covariate model, suggesting that political inclinations largely accounted for the relationships between the political climate, individual efficacy, and the use of maladaptive strategies to cope with political stressors.

In the covariate model for government efficacy, again, the significant indirect effects for adaptive coping (active coping, instrumental support, positive reframing, and religion) largely held. Moreover, two indirect effects—those involving emotional support and humor—became significant in the covariate model. Paralleling the individual efficacy covariate model, these findings suggest that the relationships between our key model variables were largely independent of political inclinations. For the maladaptive coping strategies, a more nuanced pattern emerged. While the expected indirect effect involving venting held, effects involving denial and self-distraction became nonsignificant in the covariate model. Moreover, effects involving substance abuse, self-blame, and behavioral disengagement were significant in the covariate model, but in the opposite direction than expected, such that poorer perceptions of the political environment were related to *less* substance use, self-blame, and behavioral disengagement via reduced government efficacy.

In summary, the COPE models revealed that diminished efficacy associated with perceived national decline may also have negative implications for the coping behaviors people use to contend with political stressors. Specifically, these individuals used fewer adaptive strategies to cope and, at times, more maladaptive strategies. Moreover, effects involving the adaptive strategies were generally robust to the influences of political inclinations like voter type and party affiliation, whereas the effects involving maladaptive strategies were commonly—but not wholly—accounted for by political inclinations.

## 4. Discussion

Our research suggests that Americans’ perceptions of the state of the nation may have significant implications for efficacy and well-being. Specifically, the measurement models showed that individuals who perceived that the political climate is worsening and believed that the U.S. is failing to live up to its core values also reported worse overall well-being and less effective coping with political stressors. Our structural equation models found that diminished efficacy related to the political climate may help to explain these relationships, as perceived national decline was associated with decreased perceptions that one’s voice matters in the political process (i.e., individual efficacy) and diminished perceptions of the U.S. government’s ability or willingness to meet its obligations to its people (i.e., government efficacy). In turn, decreased individual and government efficacy were each associated with worse overall well-being and diminished coping. Importantly, many of these relationships remained significant even after controlling for the effects of the participants’ 2024 presidential vote choice and political party affiliation, suggesting that regardless of whether one’s political ingroup is in power, individuals can experience psychological turmoil in the current political landscape.

The current work contributes to the existing literature in several ways. First, prior research suggests that when an individual’s preferred party is in political power, they trust their government more, report greater political competence, and feel more politically efficacious (Clarke & Acock, 1989; Davis & Hitt, 2017; Karp & Banducci, 2008; Lambert et al., 1986). On the one hand, our data converged with these prior findings: in 2025, Republicans and Trump voters tended to report greater individual and government efficacy compared to other parties or voter types. On the other hand, in our study, the relationships between concerns about the political climate and national values, efficacy, and well-being were largely robust against partisan influence. This finding is novel by demonstrating an area of convergence—rather than divergence—between partisans. In the context of intensifying political polarization and escalating interparty conflict (Abramowitz & Webster, 2016; Heltzel & Laurin, 2020), it is particularly noteworthy that the relationships between perceived societal instability, disconnection from nation-defining standards, and diminished efficacy were comparable across political groups. Despite deepening partisan divides and increasing political change, our research suggests that Americans across the political spectrum are similarly attuned to—and mutually invested in—the perceived health and stability of the nation.

This research also supplements the broader literature on the relationship between national politics and emotional and physical well-being (Chan et al., 2021; Tavits, 2008; Zimmerman et al., 1999). Political identities increasingly serve as core social identities that shape how people see themselves and others (Iyengar et al., 2019; Norman & Green, 2025). Accordingly, changes in the political climate have implications for individual functioning. In the American Psychological Association’s (2024) *Stress in America* survey, 77% of American adults—including 79% of Democrats and 80% of Republicans—reported that concerns about the nation’s future were a significant source of stress in their lives. Indeed, Americans have reported worse sleep, shorter tempers, more obsessive thoughts, and worse physical health due to politics (Smith, 2022; Neupert et al., 2021). Prior work also suggests that diminished personal control, institutional distrust, and perceptions of political dysfunction or instability undermine well-being (Aassve et al., 2024; Bandura, 1997; El Khoury-Malhame et al., 2024; Lachman & Weaver, 1998; Oishi et al., 2018; Skaff, 2007). Our findings converge with this prior evidence, suggesting that a lack of individual and government efficacy may, in part, explain the relationship between political turmoil and distress for Americans from across the political spectrum. However, given the cross-sectional design of the current study, the results provide correlational rather than causal evidence regarding this relationship.

Our findings also add to the literature on how people cope with political circumstances. Prior research suggests that tumultuous political events—such as an electoral loss or sociopolitical unrest—can trigger engagement in both maladaptive and adaptive coping strategies (DeGroot & Carmack, 2021; El Khoury-Malhame et al., 2024). In the current political climate, our models paint a more nuanced picture by illustrating how partisanship might shape—or not shape—the use of these coping strategies.

Consistent with prior research, given that the Republican party won the 2024 presidential election, one might expect that liberals would have engaged in more coping strategies than conservatives in our study. Indeed, Democrats in our sample engaged in more coping behaviors than Republicans—both adaptive and maladaptive—which is consistent with the research suggesting that an electoral loss can increase coping behaviors. However, in this sample, the relationships between perceptions of the political landscape, efficacy, and coping were at times independent of partisan influences. In particular, individuals who perceived national decline and felt diminished efficacy generally used fewer adaptive strategies overall, and these relationships persisted after accounting for the participants’ 2024 presidential vote choice and political party affiliation. These findings are novel in suggesting that current political circumstances might diminish individuals’ abilities to cope healthily with political stressors regardless of whether their chosen candidate or party is currently in power.

Conversely, poor national conditions and diminished efficacy were associated with greater use of some maladaptive strategies (e.g., denial, self-distraction, venting) and less use of others (e.g., substance use, self-blame). Aligning with prior research, many of these relationships were nullified after accounting for the participants’ 2024 presidential vote choice and party affiliation, suggesting that the use of maladaptive strategies was largely accounted for by one’s political inclinations. What is notable is that increased maladaptive coping (compared to Trump voters) was most prevalent amongst ineligible voters and eligible nonvoters, not Harris or third-party voters, leading us to speculate that individuals who refrained from voting (by choice or otherwise) may bear greater emotional burden regarding the election than those who participated in the election.

Taken together, people who are concerned about deteriorations in the political climate and the nation’s commitment to its core values are more likely to have ineffective coping responses to political stressors because current political conditions diminish their sense of individual efficacy to impact the political landscape, and lead them to question whether their government has its people’s best interests in mind. Although vote choice and party affiliation showed some associations with coping responses, our findings also suggest that perceptions of national decline and diminished political efficacy may be more proximal drivers of ineffective coping than partisanship alone. Together, these findings suggest that interventions aimed at bolstering efficacy and addressing national decline may be critical for healthier coping, beyond partisan alignment.

### Limitations and Future Directions

In light of the limitations of the current study, we recommend several avenues for future inquiry. First, the current results are timely but also cross-sectional and context dependent. In order to examine our hypotheses, we utilized a non-probability sampling platform and created custom sampling quotas based primarily on vote choice. As a result, the current sample deviates somewhat from national demographic benchmarks. For example, in our sample, Hispanic/Latino and Asian participants were underrepresented, and women and people with lower incomes were overrepresented (U.S. Census Bureau). Longitudinal research with a nationally representative sample is needed to test the stability and generalizability of these findings over time as the political landscape in the United States evolves. While historical trends have reflected long-diminishing institutional trust and political efficacy amongst the American populace (American National Election Studies, 2026; Pew Research Center, 2025), the recent acceleration of democratic backsliding (Carrier & Carothers, 2025) warrants continued inquiry to understand how this rapidly changing and unprecedented political context continues to shape efficacy and well-being into the future. Relatedly, the present work focuses on a U.S. sample at a specific historical moment characterized by rapid political change and heightened uncertainty. Although this context is relevant to our current research questions, it may constrain generalizability to other national contexts or other periods of political stability. Comparative work across national contexts could clarify whether the observed dynamics are specific to the American political landscape or reflect a more general psychological response to perceived societal deterioration.

Second, the cross-sectional nature of this work precludes strong causal inferences. For example, lower efficacy or well-being might heighten vigilance toward societal decline or amplify perceptions of institutional dysfunction, rather than result from them. We suspect that the relationships among our key model variables are recursive in nature: political instability may erode efficacy and well-being, which in turn discourages civic participation and contributes to further political deterioration. Consistent with this account, participants in our study who perceived national decline were less likely to positively reframe their circumstances and more likely to report feelings of disengagement. These responses can foster political apathy, cynicism, and disillusionment and undermine Americans’ willingness to participate in political systems, such as collective actions aimed at improving social conditions or upholding democracy (Druckman, 2024; Zhelnina, 2020).

At the same time, it is possible that the relationships described in the current research have implications for engagement in anti-democratic behavior. For example, research has documented an association between adverse mental health outcomes and support for political violence (Baum et al., 2024). Individuals are also more willing to sacrifice democratic values if they believe that doing so would enhance public safety (Chu et al., 2025) or improve societal conditions (e.g., the economy; Neundorf et al., 2026). Willingness to sacrifice democratic values is especially prevalent when political polarization and party attachment are high (Frederiksen, 2024; Graham & Svolik, 2020). Future research is needed to examine how politically induced declines in efficacy and well-being shape these collective emotions and behaviors.

Third, although we observed many statistically significant effects in our models, the effect sizes varied. According to Cohen’s (1988) benchmarks for interpreting standardized path coefficients, the relationships between political conditions and efficacy varied from small to large, and associations between efficacy and well-being were mostly small to medium. Moreover, most indirect effects were small. Small indirect effects were not entirely unexpected, given that many variables can contribute to personal well-being beyond political conditions or efficacy alone. However, these data illuminate a need to examine additional variables and how they might integrate with our model to increase explanatory power.

## 5. Conclusions

Against a backdrop of rapid political change, heightened conflict, and near-constant exposure to political information, the present findings offer timely insight into how Americans are psychologically navigating an unusually volatile political environment. At a moment when national polling indicates that most Americans believe that the United States is veering off course (AP-NORC Center for Public Affairs Research, 2025; Guskin, 2025; Harvard CAPS/Harris Poll, 2025; PRRI, 2025; YouGov, 2025), the present findings illuminate the potential psychological costs of that collective unease. Under unstable political conditions, people’s well-being and ability to handle political stressors is compromised. Across the political spectrum, perceiving that the political climate is deteriorating and believing that the U.S. is failing to live up to its foundational values may diminish individuals’ sense that they can shape political outcomes and lessen their confidence that their nation’s government is able and willing to meet the needs of its constituents. These perceptions are associated with a variety of negative outcomes, including but not limited to worse relationships, poorer health, a diminished sense of meaning and accomplishment, and less effective coping with political stressors. The results suggest that ongoing concerns about the health of American democracy are not merely abstract or ideological. These concerns are experienced at the level of everyday functioning, serving as chronic, cumulative psychological stressors undermining Americans’ well-being.

These findings also serve as a foundation toward critical action. Understanding the civic and well-being implications of political instability and perceived national decline is an essential first step for identifying ways to support Americans as they navigate political change. By clarifying how political conditions relate to eroded well-being through efficacy, this work informs efforts to strengthen civic engagement, restore a sense of agency, and promote resilience in the face of ongoing democratic uncertainty—both individually and collectively.

## Figures and Tables

**Table 1:** Sample demographics.

Measure	*N*	Percent of Sample
U.S. Census region		
Midwest	1693	23.47%
Northeast	1192	16.52%
South	2962	41.05%
West	1331	18.45%
Missing/no response	37	0.51%
Voter type (2024 Presidential election)		
Ineligible to vote	258	3.58%
Eligible to vote, but did not vote	1094	15.16%
Donald Trump (Republican Party)	2752	38.14%
Kamala Harris (Democratic Party)	2852	39.53%
Jill Stein (Green Party)	60	0.83%
Chase Oliver (Libertarian Party)	61	0.85%
Robert F. Kennedy, Jr. (Independent)	86	1.19%
Another candidate	52	0.72%
Gender identity		
Man	2727	37.80%
Woman	4387	60.80%
Transgender	11	0.15%
Non-binary	15	0.21%
Genderfluid	4	0.06%
Genderqueer	2	0.03%
Agender	1	0.01%
Two-spirit	1	0.01%
Another gender identity	6	0.08%
Prefer not to say	4	0.06%
Selected more than one gender identity	41	0.57%
Missing/no response	16	0.22%
Race or ethnicity		
White	5545	76.85%
Black or African American	882	12.22%
American Indian or Alaska Native	61	0.85%
Asian	132	1.83%
Native Hawaiian or other Pacific Islander	8	0.11%
Hispanic or Latino	166	2.30%
Middle Eastern or North African	4	0.06%
Another race or ethnicity	38	0.53%
Multiracial (selected more than one race or ethnicity)	378	5.24%
Missing/no response	1	0.01%
Annual household income, gross (USD)		
Less than $25,000	1477	20.47%
$25,000 to $49,999	1907	26.43%
$50,000 to $74,999	1378	19.10%
$75,000 to $99,999	860	11.92%
$100,000 to $149,999	937	12.99%
$150,000 or more	521	7.22%
Prefer not to say	127	1.76%
Missing/no response	8	0.11%
Political party affiliation		
Democrat	2552	35.37%
Republican	2488	34.48%
Libertarian	109	1.51%
Green Party	25	0.35%
Tea Party	9	0.12%
Socialist	26	0.36%
Democratic Socialist	60	0.83%
Independent/unaffiliated	1878	26.03%
Another political party	67	0.93%
Missing/no response	1	0.01%

**Table 2:** Structural equation model, indirect effects: Individual efficacy and PERMA (base model).

Indirect Effect	*β*	*SE*	*Z*	*p*	95% CI
POPC→individual efficacy→positive emotion	**0.20**	0.01	20.87	0.000	0.18, 0.22
BCAV→individual efficacy→positive emotion	**0.04**	0.01	7.00	0.000	0.03, 0.06
POPC→individual efficacy→engagement	**0.17**	0.01	15.61	0.000	0.15, 0.19
BCAV→individual efficacy→engagement	**0.04**	0.01	6.64	0.000	0.03, 0.05
POPC→individual efficacy→relationships	**0.14**	0.01	14.92	0.000	0.12, 0.16
BCAV→individual efficacy→relationships	**0.03**	0.01	6.55	0.000	0.02, 0.04
POPC→individual efficacy→meaning	**0.17**	0.01	19.08	0.000	0.15, 0.19
BCAV→individual efficacy→meaning	**0.04**	0.01	6.83	0.000	0.03, 0.05
POPC→individual efficacy→accomplishment	**0.23**	0.01	19.89	0.000	0.21, 0.25
BCAV→individual efficacy→accomplishment	**0.05**	0.01	6.89	0.000	0.04, 0.06
POPC→individual efficacy→negative emotion	**−0.10**	0.01	−10.20	0.000	−0.12, −0.08
BCAV→individual efficacy→negative emotion	**−0.02**	0.01	−6.16	0.000	−0.03, −0.01
POPC→individual efficacy→health	**0.16**	0.01	17.50	0.000	0.14, 0.18
BCAV→individual efficacy→health	**0.04**	0.01	6.80	0.000	0.03, 0.04
POPC→individual efficacy→happiness	**0.17**	0.01	19.39	0.000	0.15, 0.19
BCAV→individual efficacy→happiness	**0.04**	0.01	6.96	0.000	0.03, 0.05
POPC→individual efficacy→loneliness	**−0.02**	0.02	−2.84	0.005	−0.04, −0.01
BCAV→individual efficacy→loneliness	**−0.01**	0.01	−2.63	0.008	−0.01, −0.001

*Note.* POPC = Perceptions of political climate; BCAV = Beliefs about core American values. Significant effects (*p* < 0.05) are in bold.

**Table 3:** Structural equation model, indirect effects: Government efficacy and PERMA (base model).

Indirect Effect	*β*	*SE*	*Z*	*p*	95% CI
POPC→government efficacy→positive emotion	**0.23**	0.01	21.78	0.000	0.20, 0.24
BCAV→government efficacy→positive emotion	**0.03**	0.01	6.58	0.000	0.02, 0.04
POPC→government efficacy→engagement	**0.18**	0.01	15.28	0.000	0.16, 0.20
BCAV→government efficacy→engagement	**0.02**	0.003	6.21	0.000	0.01, 0.03
POPC→government efficacy→relationships	**0.14**	0.01	13.81	0.000	0.12, 0.16
BCAV→government efficacy→relationships	**0.02**	0.01	6.12	0.000	0.01, 0.02
POPC→government efficacy→meaning	**0.18**	0.01	17.86	0.000	0.16, 0.20
BCAV→government efficacy→meaning	**0.02**	0.01	6.40	0.000	0.02, 0.03
POPC→government efficacy→accomplishment	**0.24**	0.01	19.32	0.000	0.22, 0.27
BCAV→government efficacy→accomplishment	**0.03**	0.01	6.45	0.000	0.02, 0.04
POPC→government efficacy→negative emotion	**−0.12**	0.01	−10.53	0.000	−0.14, −0.10
BCAV→government efficacy→negative emotion	**−0.01**	0.01	−5.94	0.000	−0.02, −0.01
POPC→government efficacy→health	**0.17**	0.01	16.50	0.000	0.15, 0.19
BCAV→government efficacy→health	**0.02**	0.01	6.32	0.000	0.01, 0.03
POPC→government efficacy→happiness	**0.18**	0.01	19.75	0.000	0.17, 0.20
BCAV→government efficacy→happiness	**0.02**	0.01	6.53	0.000	0.02, 0.03
POPC→government efficacy→loneliness	**−0.03**	0.02	−2.54	0.011	−0.04, −0.01
BCAV→government efficacy→loneliness	**−0.003**	0.004	−2.37	0.018	−0.01, −0.001

*Note.* POPC = Perceptions of political climate; BCAV = Beliefs about core American values. Significant effects (*p* < 0.05) are in bold.

**Table 4:** Structural equation model, indirect effects: Individual efficacy and PERMA (covariate model).

Indirect Effect	*β*	*SE*	*Z*	*p*	95% CI
POPC→individual efficacy→positive emotion	**0.16**	0.01	14.81	0.000	0.14, 0.18
BCAV→individual efficacy→positive emotion	**0.04**	0.01	5.75	0.000	0.03, 0.05
POPC→individual efficacy→engagement	**0.16**	0.01	12.71	0.000	0.13, 0.18
BCAV→individual efficacy→engagement	**0.04**	0.01	5.55	0.000	0.02, 0.05
POPC→individual efficacy→relationships	**0.11**	0.01	11.03	0.000	0.09, 0.13
BCAV→individual efficacy→relationships	**0.03**	0.01	5.30	0.000	0.02, 0.04
POPC→individual efficacy→meaning	**0.14**	0.01	14.05	0.000	0.12, 0.16
BCAV→individual efficacy→meaning	**0.03**	0.01	5.63	0.000	0.02, 0.04
POPC→individual efficacy→accomplishment	**0.19**	0.01	14.84	0.000	0.17, 0.21
BCAV→individual efficacy→accomplishment	**0.04**	0.01	5.68	0.000	0.03, 0.06
POPC→individual efficacy→negative emotion	**−0.07**	0.01	−6.31	0.000	−0.09, −0.04
BCAV→individual efficacy→negative emotion	**−0.02**	0.01	−4.53	0.000	−0.02, −0.01
POPC→individual efficacy→health	**0.14**	0.01	13.37	0.000	0.12, 0.16
BCAV→individual efficacy→health	**0.03**	0.01	5.69	0.000	0.02, 0.04
POPC→individual efficacy→happiness	**0.13**	0.01	13.35	0.000	0.11, 0.15
BCAV→individual efficacy→happiness	**0.03**	0.01	5.63	0.000	0.02, 0.04
POPC→individual efficacy→loneliness	−0.01	0.02	−1.60	0.110	−0.03, 0.003
BCAV→individual efficacy→loneliness	0.00	0.01	−1.52	0.129	−0.01, 0.001

*Note.* POPC = Perceptions of political climate; BCAV = Beliefs about core American values. Significant effects (*p* < 0.05) are in bold.

**Table 5:** Structural equation model, indirect effects: Government efficacy and PERMA (covariate model).

Indirect Effect	*β*	*SE*	*Z*	*p*	95% CI
POPC→government efficacy→positive emotion	**0.21**	0.02	17.22	0.000	0.19, 0.24
BCAV→government efficacy→positive emotion	**0.03**	0.01	5.80	0.000	0.02, 0.04
POPC→government efficacy→engagement	**0.19**	0.01	13.37	0.000	0.16, 0.21
BCAV→government efficacy→engagement	**0.03**	0.004	5.49	0.000	0.02, 0.04
POPC→government efficacy→relationships	**0.13**	0.01	10.68	0.000	0.11, 0.16
BCAV→government efficacy→relationships	**0.02**	0.01	5.22	0.000	0.01, 0.03
POPC→government efficacy→meaning	**0.17**	0.01	14.24	0.000	0.15, 0.19
BCAV→government efficacy→meaning	**0.02**	0.01	5.58	0.000	0.01, 0.03
POPC→government efficacy→accomplishment	**0.24**	0.02	15.42	0.000	0.21, 0.26
BCAV→government efficacy→accomplishment	**0.03**	0.01	5.66	0.000	0.02, 0.04
POPC→government efficacy→negative emotion	**−0.09**	0.02	−6.42	0.000	−0.11, −0.06
BCAV→government efficacy→negative emotion	**−0.01**	0.01	−4.58	0.000	−0.02, −0.01
POPC→government efficacy→health	**0.17**	0.01	13.97	0.000	0.15, 0.19
BCAV→government efficacy→health	**0.02**	0.01	5.62	0.000	0.01, 0.03
POPC→government efficacy→happiness	**0.16**	0.02	14.12	0.000	0.14, 0.18
BCAV→government efficacy→happiness	**0.02**	0.01	5.62	0.000	0.01, 0.03
POPC→government efficacy→loneliness	−0.02	0.02	−1.33	0.184	−0.04, 0.01
BCAV→government efficacy→loneliness	−0.002	0.01	−1.28	0.202	−0.01, 0.001

*Note.* POPC = Perceptions of political climate; BCAV = Beliefs about core American values. Significant effects (*p* < 0.05) are in bold.

**Table 6:** Structural equation model, indirect effects: Individual efficacy and COPE (base model).

Indirect Effect	*β*	*SE*	*Z*	*p*	95% CI
POPC→individual efficacy→self-distraction	**−0.08**	0.01	−6.76	0.000	−0.10, −0.06
BCAV→individual efficacy→self-distraction	**−0.01**	0.002	−4.96	0.000	−0.02, −0.01
POPC→individual efficacy→active coping	**0.06**	0.004	6.14	0.000	0.04, 0.08
BCAV→individual efficacy→active coping	**0.01**	0.002	4.22	0.000	0.01, 0.02
POPC→individual efficacy→denial	**−0.03**	0.01	−2.64	0.008	−0.05, −0.01
BCAV→individual efficacy→denial	**−0.01**	0.001	−2.48	0.013	−0.01, −0.001
POPC→individual efficacy→substance use	**0.04**	0.01	3.93	0.000	0.02, 0.06
BCAV→individual efficacy→substance use	**0.01**	0.002	3.29	0.001	0.003, 0.01
POPC→individual efficacy→emotional support	**0.03**	0.01	3.40	0.001	0.01, 0.05
BCAV→individual efficacy→emotional support	**0.01**	0.002	2.82	0.005	0.002, 0.01
POPC→individual efficacy→instrumental support	**0.05**	0.01	4.90	0.000	0.03, 0.07
BCAV→individual efficacy→instrumental support	**0.01**	0.002	3.64	0.000	0.004, 0.01
POPC→individual efficacy→behavioral disengagement	−0.01	0.004	−0.61	0.540	−0.03, 0.01
BCAV→individual efficacy→behavioral disengagement	−0.001	0.001	−0.60	0.545	−0.01, 0.003
POPC→individual efficacy→venting	**−0.09**	0.01	−7.72	0.000	−0.11, −0.07
BCAV→individual efficacy→venting	**−0.02**	0.002	−5.18	0.000	−0.02, −0.01
POPC→individual efficacy→positive reframing	**0.10**	0.01	10.32	0.000	0.09, 0.12
BCAV→individual efficacy→positive reframing	**0.02**	0.003	5.25	0.000	0.01, 0.03
POPC→individual efficacy→planning	−0.01	0.01	−1.00	0.318	−0.03, 0.01
BCAV→individual efficacy→planning	−0.002	0.001	−0.99	0.321	−0.01, 0.002
POPC→individual efficacy→humor	0.01	0.01	1.11	0.266	−0.01, 0.03
BCAV→individual efficacy→humor	0.002	0.002	1.07	0.282	−0.002, 0.01
POPC→individual efficacy→acceptance	−0.01	0.004	−0.80	0.421	−0.03, 0.01
BCAV→individual efficacy→acceptance	−0.002	0.001	−0.80	0.422	−0.01, 0.002
POPC→individual efficacy→religion	**0.14**	0.01	14.63	0.000	0.12, 0.16
BCAV→individual efficacy→religion	**0.03**	0.004	5.77	0.000	0.02, 0.03
POPC→individual efficacy→self-blame	**0.02**	0.01	2.04	0.041	0.001, 0.04
BCAV→individual efficacy→self-blame	0.004	0.001	1.93	0.053	0.00, 0.01

*Note.* POPC = Perceptions of political climate; BCAV = Beliefs about core American values. Significant effects (*p* < 0.05) are in bold.

**Table 7:** Structural equation model, indirect effects: Government efficacy and COPE (base model).

Indirect Effect	*β*	*SE*	*Z*	*p*	95% CI
POPC→government efficacy→self-distraction	**−0.12**	0.01	−8.88	0.000	−0.15, −0.09
BCAV→government efficacy→self-distraction	**−0.01**	0.002	−5.55	0.000	−0.02, −0.01
POPC→government efficacy→active coping	**0.04**	0.01	3.48	0.001	0.02, 0.06
BCAV→government efficacy→active coping	**0.01**	0.001	2.98	0.003	0.002, 0.01
POPC→government efficacy→denial	**−0.04**	0.01	−3.38	0.001	−0.07, −0.02
BCAV→government efficacy→denial	**−0.01**	0.001	−3.08	0.002	−0.01, −0.002
POPC→government efficacy→substance use	**0.04**	0.01	3.65	0.000	0.02, 0.06
BCAV→government efficacy→substance use	**0.004**	0.001	3.12	0.002	0.002, 0.01
POPC→government efficacy→emotional support	0.02	0.01	1.75	0.080	−0.002, 0.04
BCAV→government efficacy→emotional support	0.002	0.001	1.63	0.103	0.00, 0.01
POPC→government efficacy→instrumental support	**0.05**	0.01	4.21	0.000	0.03, 0.07
BCAV→government efficacy→instrumental support	**0.01**	0.001	3.41	0.001	0.002, 0.01
POPC→government efficacy→behavioral disengagement	0.01	0.01	0.54	0.590	−0.02, 0.03
BCAV→government efficacy→behavioral disengagement	0.001	0.001	0.53	0.596	−0.002, 0.003
POPC→government efficacy→venting	**−0.14**	0.01	−11.09	0.000	−0.16, −0.11
BCAV→government efficacy→venting	**−0.02**	0.002	−5.93	0.000	−0.02, −0.01
POPC→government efficacy→positive reframing	**0.12**	0.01	10.53	0.000	0.10, 0.14
BCAV→government efficacy→positive reframing	**0.01**	0.002	5.47	0.000	0.01, 0.02
POPC→government efficacy→planning	**−0.03**	0.01	−3.01	0.003	−0.06, −0.01
BCAV→government efficacy→planning	**−0.004**	0.001	−2.79	0.005	−0.01, −0.001
POPC→government efficacy→humor	−0.001	0.01	−0.13	0.895	−0.02, 0.02
BCAV→government efficacy→humor	0.00	0.001	−0.13	0.896	−0.003, 0.002
POPC→government efficacy→acceptance	−0.02	0.01	−1.30	0.194	−0.04, 0.01
BCAV→government efficacy→acceptance	−0.002	0.001	−1.28	0.199	−0.01, 0.001
POPC→government efficacy→religion	**0.19**	0.01	17.40	0.000	0.17, 0.21
BCAV→government efficacy→religion	**0.02**	0.003	6.12	0.000	0.01, 0.03
POPC→government efficacy→self-blame	**0.04**	0.01	3.98	0.000	0.02, 0.07
BCAV→government efficacy→self-blame	**0.01**	0.001	3.39	0.001	0.002, 0.01

*Note.* POPC = Perceptions of political climate; BCAV = Beliefs about core American values. Significant effects (*p* < 0.05) are in bold.

**Table 8:** Structural equation model, indirect effects: Individual efficacy and COPE (covariate model).

Indirect Effect	*β*	*SE*	*Z*	*p*	95% CI
POPC→individual efficacy→self-distraction	−0.01	0.01	−0.54	0.590	−0.03, 0.02
BCAV→individual efficacy→self-distraction	−0.001	0.001	−0.53	0.594	−0.01, 0.003
POPC→individual efficacy→active coping	**0.08**	0.004	7.58	0.000	0.06, 0.10
BCAV→individual efficacy→active coping	**0.01**	0.002	4.14	0.000	0.01, 0.02
POPC→individual efficacy→denial	0.001	0.01	0.10	0.924	−0.02, 0.02
BCAV→individual efficacy→denial	0.00	0.001	0.09	0.925	−0.004, 0.004
POPC→individual efficacy→substance use	**0.05**	0.01	4.90	0.000	0.03, 0.07
BCAV→individual efficacy→substance use	**0.01**	0.002	3.55	0.000	0.004, 0.01
POPC→individual efficacy→emotional support	**0.06**	0.01	6.07	0.000	0.04, 0.09
BCAV→individual efficacy→emotional support	**0.01**	0.002	3.72	0.000	0.01, 0.02
POPC→individual efficacy→instrumental support	**0.07**	0.01	6.06	0.000	0.04, 0.09
BCAV→individual efficacy→instrumental support	**0.01**	0.002	3.71	0.000	0.01, 0.02
POPC→individual efficacy→behavioral disengagement	0.01	0.01	0.69	0.490	−0.01, 0.03
BCAV→individual efficacy→behavioral disengagement	0.001	0.001	0.67	0.504	−0.003, 0.01
POPC→individual efficacy→venting	−0.01	0.01	−0.90	0.369	−0.03, 0.01
BCAV→individual efficacy→venting	−0.002	0.001	−0.88	0.377	−0.01, 0.002
POPC→individual efficacy→positive reframing	**0.09**	0.01	8.14	0.000	0.07, 0.11
BCAV→individual efficacy→positive reframing	**0.02**	0.003	4.25	0.000	0.01, 0.02
POPC→individual efficacy→planning	**0.02**	0.01	2.41	0.016	0.004, 0.04
BCAV→individual efficacy→planning	**0.004**	0.002	2.08	0.038	0.00, 0.01
POPC→individual efficacy→humor	**0.04**	0.01	4.04	0.000	0.02, 0.06
BCAV→individual efficacy→humor	**0.01**	0.002	3.16	0.002	0.003, 0.01
POPC→individual efficacy→acceptance	0.02	0.01	1.53	0.125	−0.01, 0.04
BCAV→individual efficacy→acceptance	0.003	0.001	1.39	0.165	−0.001, 0.01
POPC→individual efficacy→religion	**0.07**	0.01	7.12	0.000	0.05, 0.09
BCAV→individual efficacy→religion	**0.01**	0.003	3.99	0.000	0.01, 0.02
POPC→individual efficacy→self-blame	0.01	0.01	0.48	0.634	−0.02, 0.03
BCAV→individual efficacy→self-blame	0.001	0.002	0.47	0.641	−0.003, 0.01

*Note.* POPC = Perceptions of political climate; BCAV = Beliefs about core American values. Significant effects (*p* < 0.05) are in bold.

**Table 9:** Structural equation model, indirect effects: Government efficacy and COPE (covariate model).

Indirect Effect	*β*	*SE*	*Z*	*p*	95% CI
POPC→government efficacy→self-distraction	−0.02	0.01	−1.52	0.130	−0.05, 0.01
BCAV→government efficacy→self-distraction	−0.003	0.001	−1.49	0.135	−0.01, 0.001
POPC→government efficacy→active coping	**0.07**	0.01	5.28	0.000	0.05, 0.10
BCAV→government efficacy→active coping	**0.01**	0.002	3.76	0.000	0.004, 0.01
POPC→government efficacy→denial	0.004	0.01	0.24	0.806	−0.03, 0.03
BCAV→government efficacy→denial	0.00	0.001	0.24	0.811	−0.003, 0.004
POPC→government efficacy→substance use	**0.07**	0.01	5.29	0.000	0.04, 0.10
BCAV→government efficacy→substance use	**0.01**	0.002	3.82	0.000	0.01, 0.01
POPC→government efficacy→emotional support	**0.08**	0.01	6.29	0.000	0.06, 0.11
BCAV→government efficacy→emotional support	**0.01**	0.002	4.05	0.000	0.01, 0.02
POPC→government efficacy→instrumental support	**0.10**	0.01	7.01	0.000	0.07, 0.12
BCAV→government efficacy→instrumental support	**0.01**	0.002	4.30	0.000	0.01, 0.02
POPC→government efficacy→behavioral disengagement	**0.04**	0.01	2.80	0.005	0.01, 0.07
BCAV→government efficacy→behavioral disengagement	**0.01**	0.001	2.42	0.015	0.001, 0.01
POPC→government efficacy→venting	**−0.04**	0.01	−2.72	0.006	−0.07, −0.01
BCAV→government efficacy→venting	**−0.01**	0.001	−2.59	0.010	−0.01, −0.001
POPC→government efficacy→positive reframing	**0.12**	0.01	9.03	0.000	0.10, 0.15
BCAV→government efficacy→positive reframing	**0.02**	0.002	4.83	0.000	0.01, 0.02
POPC→government efficacy→planning	0.01	0.01	0.51	0.613	−0.02, 0.03
BCAV→government efficacy→planning	0.001	0.001	0.49	0.624	−0.002, 0.004
POPC→government efficacy→humor	**0.05**	0.01	3.87	0.000	0.03, 0.07
BCAV→government efficacy→humor	**0.01**	0.002	3.15	0.002	0.002, 0.01
POPC→government efficacy→acceptance	0.03	0.01	1.70	0.089	−0.004, 0.05
BCAV→government efficacy→acceptance	0.003	0.001	1.56	0.119	−0.001, 0.01
POPC→government efficacy→religion	**0.12**	0.01	10.08	0.000	0.10, 0.15
BCAV→government efficacy→religion	**0.02**	0.003	4.97	0.000	0.01, 0.02
POPC→government efficacy→self-blame	**0.03**	0.01	2.23	0.025	0.004, 0.06
BCAV→government efficacy→self-blame	**0.004**	0.002	2.04	0.041	0.00, 0.01

*Note.* POPC = Perceptions of political climate; BCAV = Beliefs about core American values. Significant effects (*p* < 0.05) are in bold.

## Data Availability

Data can be found within the Supplementary Materials in the Open Science Framework (OSF) repository. https://osf.io/x3gu6/overview?view_only=fabeeaae4de24c94808731fca8bbe8c4.

## Notes

1 In our study, the Accomplishment subscale only contained two items rather than three. One item was erroneously omitted during the initial data collection. The item was reinstated partway through data collection, but to analyze it would have significantly limited our overall sample size to approximately *n* = 2000. Analyses using this item are included in the Supplementary Materials.

2 Estimating the direct effects of our predictors on our outcomes produced statistical suppression effects, particularly in the models involving government efficacy. For ease of interpretation and comparison, we report models where these direct effects are constrained to zero. Models that freely estimated these direct effects can be found in the Supplementary Materials.

## Supplementary Materials

Supplementary materials can be found in the Open Science Framework (OSF) repository. https://osf.io/x3gu6/overview?view_only=fabeeaae4de24c94808731fca8bbe8c4.
